# Draft Genome Sequence of the Bacterium Paraburkholderia aromaticivorans AR20-38, a Gram-Negative, Cold-Adapted Degrader of Aromatic Compounds

**DOI:** 10.1128/MRA.00463-20

**Published:** 2020-07-02

**Authors:** Caroline Poyntner, Dechao Zhang, Rosa Margesin

**Affiliations:** a Institute of Microbiology, University of Innsbruck, Innsbruck, Austria; b Institute of Oceanology, Chinese Academy of Sciences, Qingdao, China; c University of Chinese Academy of Sciences, Beijing, China; University of Maryland School of Medicine

## Abstract

Here, we report the draft genome sequence of Paraburkholderia aromaticivorans strain AR20-38, a cold-adapted Gram-negative bacterium. It was isolated from Alpine forest soil and can degrade a range of aromatic compounds.

## ANNOUNCEMENT

*Paraburkholderia* is a genus of *Proteobacteria*, class *Betaproteobacteria*. Members of this genus have been isolated from diverse ecological niches, including pristine and contaminated soil, sediments, rocks, and plants (1, 2).

Paraburkholderia aromaticivorans strain AR20-38 was isolated from an Italian Alpine forest soil sample (3). Soil samples were surface spread onto Reasoner’s 2A (R2A) agar. Growing strains were subcultured, purified, and stored at −80°C. Due to its properties, strain AR20-38 was chosen for full-genome sequencing.

The strain was grown from a single colony on R2A agar and was further inoculated in nutrient broth incubated at 10°C until the stationary growth phase. After lyophilization, genomic DNA was extracted using lysozyme, SDS, and phenol-chloroform-isoamyl alcohol. DNA quality and quantity were determined using a Qubit 2.0 fluorometer (Thermo Fisher Scientific) and agarose gel electrophoreses. DNA was used for Oxford Nanopore and Illumina sequencing.

The one-dimensional (1D) ligation sequencing kit (SQK-LSK109 kit; Oxford Nanopore) was used with additional reagents from New England Biolabs (NEBNext FFPE repair mix, NEBNext end repair/dA-tailing module, and NEBNext quick ligation module) following the manufacturer’s recommendations. No size selection or shearing was applied.

For Illumina sequencing, 1 μg DNA was used with the NEBNext Ultra DNA library prep kit (New England Biolabs) following the manufacturer’s recommendations. The Nanopore library was sequenced on the PromethION instrument (PromethION flow cells, FLO-PRO002; Oxford Nanopore), and the Illumina library was sequenced on the Illumina NovaSeq PE150 instrument at the Beijing Novogene Bioinformatics Technology Co. Ltd.

For all software used, default parameters were used except where otherwise noted.

The Nanopore fast5 file was base called using Guppy (Oxford Nanopore), and qcat was applied. Nanopore quality control was achieved using NanoPlot with a threshold value (Q) of >7, resulting in 132,813 reads with a median read length of 15,994 bp and an *N*_50_ value of 19,781 bp. Illumina data were quality controlled using Readfq, which removed reads containing more than 40% low-quality bases (quality value, ≤20), overlaps with adapter sequences, and duplicates. The Illumina reads were assembled using SPAdes 3.10.0 (4). A hybrid assembly was created using Racon (5), miniasm (6), and Unicycler 0.4.7 (7). The contigs were controlled for overlapping end sequences and start, end, *dnaA*, and *repA* sites, resulting in three assembled, circular chromosomes and one plasmid (Table 1). GeneMarkS 4.17 (8), RepeatMasker 4.0.5 (9), and Tandem Repeats Finder (TRF) 4.07b (10) were used to predict coding genes, interspersed repetitive sequences, and tandem repeats. Further, tRNA genes were predicted using tRNAscan-SE 1.3.1 (11), rRNA genes were predicted using RNAmmer 1.2 (12), and snRNA genes were predicted using the Rfam database (13). The assembled genome contained genomic islands (IslandPath 0.2 [14]), prophage sequences (phiSpy 2.3 [15]), and CRISPRs (CRISPRdigger 1.0 [16]).

**TABLE 1:** Genome data of the three chromosomes and the plasmid

Chromosome or plasmid	Size (bp)	GC content (%)	Form	No. of rRNA genes
Chromosome 1	2,486,079	60.09	Circular	3
Chromosome 2	3,638,240	62.46	Circular	9
Chromosome 3	4,573,438	62.5	Circular	9
Plasmid	142,975	59.48	Circular	

Gene functions were determined using Gene Ontology (GO) (17, 18), KEGG (19, 20), COG (21), the transporter classification database (TCDB) (22), and SWISS-PROT (23). Additional secretory proteins (SignalP 4.1 [24]), type I to VII proteins (EffectiveT3 [25]), and secondary metabolism gene clusters (antiSMASH 2.0.2 [26]) were predicted. PHI (27), VFDB (28), ARDB 1.1 (29), and CAZy (30) were applied. The results are in line with properties observed in the lab.

### Data availability.

The assembled genome and sequencing reads have been deposited in GenBank under the BioProject number PRJNA624061 and the accession numbers CP051514, CP051515, CP051516, and CP051517 and in the NCBI Sequence Read Archive under the numbers SRX8492130 and SRX8492131.
